# Nutritional considerations for athletes with diabetes: optimizing performance and glycemic control

**DOI:** 10.3389/fnut.2026.1737219

**Published:** 2026-03-27

**Authors:** Liguang Xu, Wei Zhang

**Affiliations:** Wuhan Sports University, Wuhan, Hubei, China

**Keywords:** athletes, carbohydrate timing, continuous glucose monitoring (CGM), glycemic control, nutrition, performance optimization, type 1 diabetes, type 2 diabetes

## Abstract

Athletes with diabetes encounter the intricate physiological challenge of harmonizing optimal physical performance with meticulous glycemic control. Although nutritional management is crucial for success, generic guidelines often do not provide the required differentiation for various diabetes causes and contemporary insulin delivery methods. This narrative review aims to consolidate the latest research on optimizing macronutrient intake, hydration, and micronutrient support specifically for athletes with Type 1 (T1D) and Type 2 (T2D) diabetes. A structured search of the literature was conducted on Google Scholar (2015–2025) to identify relevant peer-reviewed clinical trials, meta-analyses, and expert consensus statements. The identified nutritional strategies were then analyzed and classified based on an evidence-grading framework: Level A (Strong evidence/Meta-analyses), Level B (Moderate evidence/Single RCTs), and Level C (Expert consensus). Carbohydrate timing and dosing are crucial factors in maintaining normal blood sugar levels during exercise, and they need to be adjusted based on the intensity and duration of the activity, as well as the type of insulin therapy being used (e.g., multiple daily injections vs. automated insulin delivery systems). This review presents structured guidelines for managing carbohydrate intake before, during, and after exercise, highlighting the importance of protein for muscle recovery and the influence of micronutrients like magnesium and vitamin D on metabolic function. Additionally, the use of Continuous Glucose Monitoring (CGM) data is discussed as a valuable tool for reducing fluctuations in blood sugar levels and preventing exercise-induced hypoglycemia. Optimizing athletic performance in individuals with diabetes necessitates a comprehensive, multidisciplinary strategy. By coordinating dietary choices with appropriate treatment modalities and utilizing evidence-based assessments, healthcare providers can offer more secure and efficient recommendations for both competitive and recreational athletes.

## 1 Introduction

Exercise is the cornerstone of diabetes treatment, along with medical nutrition therapy. According to the American Diabetes Association (1) (ADA) Standards of Medical Care in Diabetes, persons with diabetes should engage in resistance training as well as aerobic exercise. The American Diabetes Association (ADA) recently released revised guidelines and safety measures for exercise and physical activity for those with type 1 diabetes, type 2 diabetes (2). In order to minimize consecutive days without activity, they stipulate that this should involve at least 150 min of moderate-to-vigorous aerobic activity each week, distributed across at least 3 days, and two to three resistance training sessions per week on days that aren’t continuous (3). Regular exercise is associated with improved glucose management and insulin sensitivity, reduced blood pressure, avoided or minimized weight gain, and improved lipid profile all of which are independent risk factors for the development of type 2 diabetes (4).

Numerous professional athletes who compete in various sports have diabetes. To keep them healthy and guarantee their best strength and performance, athletes with diabetes should modify their diet in accordance with the recommendations made for the general public (5), perhaps paying particular consideration of the daily meal quantity and nutritional content. For athletes with diabetes, nutritional management necessitates a thorough evaluation that covers a number of important areas: (1) early assessment according to the sport played, including the kind of exercise (e.g., strength or endurance) (6); (2) blood glucose testing and optimization of glycemic levels attained while training; (3) careful dietary intake monitoring and antidiabetic medication (7).

The American Diabetes Association (ADA) recommended a diet that includes a variable amount of CHO, ranging from 45 to 55 percent of total calories (8). Although eating foods with a low glycemic index (GI) can lower postprandial glycemia, several studies have demonstrated that consuming sucrose does not raise glycemic levels more than starch (9). Although it is advised to limit added sugars to 30 g per day, they are not strictly prohibited, especially in the context of exercise (10). Other vital macronutrients like proteins and fats must be included in the diet of athletes with diabetes (11) because, when consumed in moderation, they may marginally raise postprandial blood glucose levels (12) and increase the demand for prandial insulin.

According to the current guidelines, individuals with diabetes should consume no more than 15% of their daily calories from protein, assuming that they consume 0.80–1 g of protein per kilogram of body weight (13). Polyunsaturated fatty acids (PUFA) should make up 10% of total calories (14), while fat content might range from 30 to 50% (15). Consuming fish and olive oils is advised since they are important sources of omega-3 (*ω*-3) and monounsaturated fatty acids (MUFA), respectively (11).

While there are general frameworks available, there is a crucial requirement for stratified guidance that differentiates between Type 1 (T1D) and Type 2 (T2D) diabetes and takes into consideration modern therapeutic technologies. This narrative review is intended to consolidate current evidence on substrate management and exercise physiology in diabetes. By presenting structured algorithms for pre-, during-, and post-exercise nutrition, this review aims to provide a practical, evidence-based framework to enhance both athletic performance and metabolic safety.

## 2 Methods

### 2.1 Search strategy

This study is a narrative review aimed at consolidating the latest evidence on sports nutrition for athletes with diabetes. A thorough literature search was carried out using the Google Scholar database to locate pertinent peer-reviewed articles published between January 2015 and January 2025.

The search strategy utilized a combination of primary and secondary terms. Primary keywords included “Type 1 Diabetes,” “Type 2 Diabetes,” “Sports Nutrition,” “Athletes,” and “Exercise Physiology.” Secondary keywords encompassed “Carbohydrate Periodization,” “Glycemic Control,” “Continuous Glucose Monitoring (CGM),” “Insulin Pump Therapy (CSII),” “Automated Insulin Delivery (AID),” and “Post-exercise Recovery.”

### 2.2 Selection criteria

To maintain focus on clinical nutrition for the athletic population, the review followed specific criteria for selecting evidence:

*Inclusion criteria (included sources)*

*Peer-reviewed research:* Original studies, systematic reviews, meta-analyses, and official clinical guidelines (e.g., ADA, ISPAD).

*Target Population:* Competitive athletes or highly active individuals with Type 1 or Type 2 diabetes.

*Nutritional focus:* Studies with detailed information on macronutrients (carbohydrates, proteins, fats), insulin adjustments, or glycemic outcomes during physical activity.

*Exclusion criteria*

*Single-patient case reports:* These studies have limited applicability to the broader athletic population.

*Sedentary populations:* Studies that exclusively focus on inactive individuals or those who do not engage in regular exercise.

*General diabetes studies:* Research that addresses diabetes management without incorporating an exercise or athletic element.

*Language and source:* Non-English publications and “grey literature” such as non-peer-reviewed blogs or magazine articles.

### 2.3 Evidence grading and strength of recommendations

To address the need for clinical applicability, a grading system has been introduced to assess the strength of the recommendations presented in this review. This framework enables healthcare professionals to evaluate the credibility of the advice based on the underlying study methodology:

*Grade A (strong):* Recommendations backed by high-quality meta-analyses, systematic reviews, or multiple randomized controlled trials (RCTs).

*Grade B (moderate):* Recommendations supported by individual RCTs, large prospective cohort studies, or well-conducted observational research.

*Grade C (expert consensus):* Recommendations derived from expert committee reports, professional consensus statements (e.g., ADA, ISPAD), Narrative reviews and clinical expertise in the absence of robust experimental data.

## 3 Diabetes and exercise: physiological considerations

### 3.1 Exercise and glucose metabolism in diabetes

Exercise can help with glucose management because it increases the elimination of glucose and improves insulin action. Insulin-dependent and independent pathways mediate glucose absorption through muscle contraction and contraction-mediated skeletal muscle blood flow. Although other variables that impact systemic glucose metabolism may also have an impact, exercise-mediated glucose disposal can lower blood glucose levels. To better understand how exercise affects glucose clearance, it is necessary to take into account the components of glucose elimination. By quickly inducing the movement of glucose transporter 4 (GLUT4), muscular contraction enhances the quicker delivery of glucose into the muscle. Additionally, contraction increases blood flow to the skeletal muscles, which accelerates the rate at which glucose diffuses into the muscle interstitial space (16). GLUT4 is also drawn to the region of the muscle by insulin. In the course of exercise, exogenous glucose and muscle glycogen reserves are used up, creating a gradient of glucose/glucose-6-phosphate that promotes more glucose entry into the skeletal muscle. Based on these factors and other molecular changes in skeletal muscle signaling, exercise can impact the equilibrium of glucose for as long as 48 h (17). GLUT4 expression in skeletal muscle, insulin receptor signaling, and oxidative capacity are all improved by exercise training to optimize the action of insulin, glucose oxidation, and storage (18). Thus, Individuals with type 2 diabetes usually have higher insulin sensitivity when they engage in frequent, moderate exercise (19).

### 3.2 Exercise and insulin sensitivity

Exercise enhances insulin-independent processes that increase muscle glucose uptake during contractions and increases skeletal muscle insulin sensitivity after exercise, both of which contribute to improved metabolic management (20). Individuals with type 2 diabetes who exhibit normal glucose absorption in their skeletal muscles during exercise and appear to exhibit normal improvements in insulin sensitivity after exercise, despite their muscle tissue not responding normally to insulin, underscore the intricacy of the exercise- caused an improvement in insulin sensitivity (21).

Exercise is known to affect insulin sensitivity by enhancing the absorption of glucose. In particular, blood catecholamine rises quickly during muscular contraction, and glucose mobilization outpaces peripheral glucose absorption. This suggests that additional processes are engaged in the mobilization of hormones and substrates to sustain euglycemia during vigorous exercise (22).

One of the most well-known of the many insulin-sensitizing changes induced by prolonged exercise is increased oxidative capacity. In fact, a high mitochondrial density in skeletal muscle (SM) has been consistently emphasized as a clear characteristic of SM-IS (insulin sensitivity) (23, 24). On the other hand, loss of SM mass has been linked to mitochondrial malfunction. Additionally, an effective glucose metabolism is positively correlated with peripheral oxidative phosphorylation capacity. In summary, because of a higher total substrate utilization, among the best markers of entire-body insulin sensitivity is thought to be elevated peripheral oxidative ability (25).

### 3.3 Hypoglycemia and hyperglycemia risks

For individuals with diabetes on insulin or sulfonylureas, or other glucose-lowering drugs, exercise can be extremely risky because it can raise their risk of hypoglycemia. For diabetics, hypoglycemia and the risk of low blood glucose levels during training are significant and genuine concerns. In people with T1D, exercise can impair compensatory responses and result in hypoglycemia during the night, and episodes of severe (and especially nocturnal) hypoglycemia are linked to significant increases in mortality (26), making these factors particularly pertinent to those with T1D. Those with T2D on insulin or sulfonylurea are also at risk, though not as much (27). Exercise significantly raises the metabolic demand for glucose and increases the movement and expression of GLUT4 (28), which enhances the effects of insulin. These elements maximize the risk of hypoglycemia. For up to 48 h, exercise can affect glucose homeostasis (17). The main reason why patients with T1D avoid exercising is their fear of hypoglycemia.

The more the duration and the intensity of the physical exercise, the more skeletal muscle absorbs blood glucose. When there is moderate activity, the decrease in muscle glucose uptake-induced circulating glucose is accompanied by a reduction in plasma insulin and an increase in counterregulatory hormones, especially glucagon, which aid in glucose mobilization (29). According to a study, aerobic exercise results in a greater initial drop in blood glucose but a somewhat less prolonged hypoglycemic impact, while resistance training typically causes an acute increase in blood glucose followed by an increase in insulin sensitivity. Resistance training, however, was linked to a generally lower level of blood glucose fluctuation after exercise (30).

Increased levels of physical activity (PA) are linked to improved condition-specific health outcomes in individuals with type 1 diabetes (T1D) (31). But only about one-third of T1D patients reach the suggested PA goals (32). Concern of low blood sugar, which can lead to dangerous outcomes like seizures or unconsciousness, as well as uncomfortable symptoms like lightheadedness and impairment of physical and cognitive ability, is the most frequent obstacle to PA for persons with T1D (33).

Prior to and during aerobic activity, the suggested goal blood glucose concentration (BGC) is greater (7.0–10.0 mmol/L) than it is during non-exercise (4.0–7.0 mmol/L) (34). A counter regulatory response during resistance exercise or high-intensity interval training (HIIT) (35), may cause BGC to stay constant or even increase, particularly when done while fasting (34).

However, when done postprandial, all types of aerobic exercise, including HIIT, lower BGC (36). People with T1D may lower their pre-exercise insulin dosages and/or ingest more carbs before exercising in order to lower their risk of hypoglycemia. This technique may be especially common in endurance sports (37).

Therefore, individuals with T1D frequently experience acute hyperglycemia during exercise. Although mild to moderate hyperglycemia (e.g., BGC 7.1–13.9 mmol/L) (34) is typically regarded as acceptable for exercise, it is uncertain whether increases in blood glucose at these levels have an impact on exercise performance. Acute hyperglycemia increases water and mineral losses by diuresis of osmotic fluid and modifies immunological, endocrine, and metabolic responses to exercise. Since beta-endorphins are expected to impede performance in tests of maximal aerobic or anaerobic capacity, it may also worsen the greater rates of perceived exertion (RPE) that patients with T1D experience (38).

Furthermore, individuals with T1D may not exhibit any symptoms during mild to moderate hyperglycemia, particularly if their blood glucose levels are consistently high. Since these people have hypoglycemic symptoms and BGCs in the euglycemic range (4.0–7.0 mmol/L), hyperglycemia may help them perform better. Because acute hyperglycemia stimulates the brain’s glucose-sensing neurons, it may also lessen the central inhibition of further exercise when exhausted (39, 40). The fear of hypoglycemia may be lessened in T1D athletes when hyperglycemia is present, boosting their confidence to execute their maximum physical effort.

### 3.4 Mechanism

#### 3.4.1 Insulin signaling pathways and exercise

For insulin signaling to occur within insulin-sensitive tissues, the hormone must bind to a specific membrane receptor known as the insulin receptor, which is composed of four subunits: two extracellular *α*-subunits and two transmembrane *β*-subunits. When insulin binds to the α-subunits, it activates the intrinsic kinase activity of the β-subunits, leading to auto-phosphorylation of tyrosine residues in the receptor’s intracellular domain (41). Members of the insulin receptor substrate (IRS) family, including IRS-1, IRS-2, IRS-3, and IRS-4, are among the downstream targets that are phosphorylated as a result of this activation, which also attracts intermediate Protein molecules (42). After being phosphorylated, IRS-1 and IRS-2 attach to and activate proteins that contain the Src homology-2 (SH2) domain, including phosphoinositide 3-kinase (PI3K). The SH2 domain, approximately 100 amino acids in length, specifically recognizes and attaches to phosphorylated tyrosine residues, propagating the insulin signaling cascade. Following this, PI3K forms phosphatidylinositol (3,4,5)-trisphosphate (PI3P), a binding site of phosphoinositide-dependent kinase (PDK) (43). Atypical protein kinase C (aPKC) and one of the isoforms of protein kinase B (PKB), sometimes referred to as Akt, are activated by PDK (44). There is proof that aPKC plays a crucial role in insulin-stimulated glucose transport in skeletal muscle; exercise appears to enhance its activation, which appears to be impaired in insulin resistance (45).

The aPKC lambda/iota isoform has demonstrated a significant function in glucose transport among the other aPKC isoforms. By phosphorylating the double C2-like domain-containing protein (DOC2b), which regulates the soluble N-ethylmaleimide-sensitive factor attached protein receptor (SNARE), this enzyme makes it easier for syntaxin-4 to connect with GLUT4-containing vesicles and encourages their bonding with the plasma membrane (46). Other PKC isoforms, such as PKC*α* and PKCθ, which are triggered by the rise in intracellular calcium, are also implicated in GLUT4 translocation in addition to aPKC (47).

Researches from animal models has helped to grasp the mechanisms behind the potentiation of insulin’s effect by both acute aerobic exercise and chronic training sessions. Through additional pathways involving AMP-activated protein kinase (AMPK), a crucial enzyme triggered by muscle contraction, PE can enhance the muscle’s uptake of glucose. AMPK is a heterotrimeric protein that has the following isoforms: β1, β2, γ1, γ2, and γ3. It is made up of two regulatory subunits (beta and gamma) and a catalytic subunit (alpha). It is triggered by the phosphorylation of a threonine-172 residue in the α subunit’s activation loop (48). An energy imbalance brought on by muscle contraction may induce AMPK to become activated. Liver kinase B1 (LKB1) is now thought to be the primary protein implicated in AMPK phosphorylation among the proteins that regulate AMPK (49). Exercise-induced activation of AMPK and LKB1 has been extensively shown in both humans and animals.

Importantly, AMPK-stimulated glucose transport seems to be driven by several mechanisms, such as increased intracellular concentrations of Ca^(++)^ and bradykinin (a plasma polypeptide that causes vasodilation); increased activity of endothelial nitric oxide synthase (which increases vasodilation and the availability of nitric oxide); activation of Ca(2+)/calmodulin-dependent protein kinase (CaMK); activation of protein kinase C (PKC); and even hypoxia. For GLUT4 to translocate effectively and allow glucose to enter cells, all of these conditions must be met (41).

#### 3.4.2 Glucose transport and utilization in skeletal muscle

The aforementioned routes encourage increased GLUT4 translocation and, as a result, skeletal muscle glucose absorption. Acute increases in muscular contraction set off metabolic processes that boost the muscle’s absorption of glucose. Increased insulin sensitivity (50) and the type 4 glucose transporter’s (GLUT4) cell surface translocation, which occurs independently of insulin administration, are the two main causes of this. Furthermore, physical exercise (PE) lowers the inflammatory state, particularly by the production of anti-inflammatory cytokines and a reduction in total lipid profile, and persistently raises intramuscular GLUT4 concentration (51).

Additionally, it has been shown that AMPK activation in skeletal muscle can enhance lipid oxidation, which in turn can adapt glycogen resynthesize to PE (by preserving muscle glycogen) by inducing muscular contraction (52). In adipose tissue, certain myokines, such as interleukin-15 (IL-15) and interleukin-6 (IL-6), enhance GLUT4 expression, which can enhance PE-induced glucose absorption (53) and trigger AMPK and GLUT4 translocation to the cell surface (54).

## 4 Clinical management of type 1 diabetes among athletes

People with type 2 diabetes typically feel less concerned about hypoglycemia when exercising, unless they are using insulin or other medications that have a recognized hypoglycemic impact. On the other hand, to prevent hypoglycemia and decreased exercise participation (R. (55)), individuals with type 1 diabetes should constantly check their blood sugar levels before and after exercise, especially in relation to meals and/or insulin therapy (56, 57). A person’s safe blood glucose levels at the beginning of exercise will vary (58). Starting glucose levels between 7 mmoL/L 10.0 mmol/L are typically sufficient to offset the risk of hypoglycemia. In some circumstances where there is a greater fear of hypoglycemia, higher glucose doses may be appropriate. In certain situations where there is a greater risk of hypoglycemia, reduction in insulin doses may be appropriate. However, persons with T1DM have trouble controlling their blood glucose levels. The modality, duration, intensity, initial blood glucose level, level of fitness, and amount of insulin in circulation are some of the variables that will influence the change in blood glucose (59, 60). Given counterregulatory mechanisms that raise blood glucose, initial blood glucose ranges for weightlifting or high intensity interval training (HIIT) may be lower than those for continuous aerobic activity (e.g., 5.0–7.0 mmol/L).

### 4.1 Managing type 1 diabetes by insulin analogues delivery and AID system

Effective management of athletes with type 1 diabetes needs a thorough comprehension of the pharmacokinetics of rapid-acting insulin analogues, like Lispro and Aspart. These analogues serve as the core foundation for contemporary Automated Insulin Delivery (AID) systems because they have a quicker onset and shorter duration than human insulin, enabling the algorithm to make the quick modifications required during physical exertion. This AID system which uses Continuous subcutaneous insulin infusion utilizing a hybrid is now used by many T1DM patients who utilize insulin pumps. These devices have a closed-loop algorithm that modifies the insulin dosage according to CGM data. For T1DM, many of these systems offer a “activity” or “training” mode that increases the desired level of glucose and lowers the percentage of insulin supply. Based on the blood glucose algorithm, the AID system’s algorithm frequently administers more insulin via basal or bolus. To halt the extra insulin that the AID algorithm delivers, some patients find it helpful to switch the pump from automated to manual mode with a 50 to 80% basal reduction 90 min before exercise. Significantly, individuals with AID systems could still need to eat carbs before or during exercise. However, as the AID might offer more insulin when blood glucose starts to rise after consuming carbs without bolus coverage, it is recommended to avoid eating more than half an hour before exercise (61).

## 5 Clinical management of type 2 diabetes among athletes

Sodium-glucose cotransporter type 2 inhibitors (SGLT2is) are used as glucose-lowering agents in patients with type 2 diabetes mellitus (T2DM), either as a standalone treatment or in combination with other antidiabetic medications. The main mechanism of action of SGLT2is is the inhibition of glucose reabsorption in the kidneys, leading to increased glucose excretion in the urine and a direct reduction in blood glucose levels. This simple yet effective approach to managing T2DM is independent of insulin, reducing the risk of hypoglycemia (62).

Additionally, the glucose-lowering effects of SGLT2is are not dependent on the level of insulin secretion or insulin resistance, making them suitable for patients with T2DM and comorbid conditions such as severe obesity. By lowering hyperglycemia, SGLT2is may also indirectly address two key defects in T2DM: pancreatic *β*-cell dysfunction and insulin resistance (63) (Grade C).

In a network meta-analysis of randomized controlled trials (RCTs), all SGLT2 inhibitors (SGLT2is) used as monotherapy were found to be significantly more effective than placebo in reducing HbA1c levels from baseline (mean differences ranged from −0.59 to −1.23%), increasing the proportion of patients achieving target HbA1c levels (odds ratios ranged from 2.18 to 3.45), and reducing mean body weight (mean differences ranged from −1.63 to −3.00 kg) (64) (Grade A). Similar findings were reported in another meta-analysis specifically focusing on RCTs involving dapagliflozin as monotherapy (65) (Grade A).

### 5.1 Combination with other drugs

The combination of SGLT2 inhibitors and metformin resulted in a significantly higher reduction in HbA1c, fasting plasma glucose (FPG), body weight, and systolic blood pressure compared to metformin alone (*p* < 0.00001) (66). In four randomized controlled trials (RCTs) that assessed initial combination therapy for type 2 diabetes mellitus (T2DM), the SGLT2 inhibitor-metformin combination showed greater reductions in HbA1c and body weight compared to metformin monotherapy and SGLT2 inhibitors used alone (67) (Grade A).

Compared to a placebo, the addition of SGLT2 inhibitors to a background therapy of sulfonylurea (with or without metformin) significantly reduced HbA1c, fasting plasma glucose (FPG), and body weight after 18–24 weeks and 52–78 weeks of treatment (*p* < 0.00001) (68) (Grade A).

When added to insulin therapy in patients with type 2 diabetes mellitus (T2DM), SGLT2 inhibitors were associated with statistically significant reductions in HbA1c, FPG, and body weight. Interestingly, these effects were observed while the mean daily insulin dose was reduced by −8.79 IU. There were no statistically significant increases in the risks of all-type hypoglycemia and severe hypoglycemic episodes (69) (Grade A).

Clinical data has demonstrated that the effectiveness of combined SGLT2 inhibitor (SGLT2i) and GLP-1 receptor agonist (GLP1RA) therapy is partially additive in reducing HbA1c levels, body weight, and systolic blood pressure (70). These positive effects were observed when an SGLT2i was added to a GLP1RA, when a GLP1RA was added to an SGLT2i (71), or when the initial combination was compared to each individual therapy (72). From an endocrine perspective, SGLT2 inhibitors tended to increase serum glucagon levels, while GLP-1 receptor agonists reduced glucagon secretion (73) (Grade C).

### 5.2 Side effects of the newer drugs

SGLT2 inhibitors (SGLT2i), such as empagliflozin and dapagliflozin, provide glycemic control without relying on insulin. However, they pose three significant risks for athletes: hypovolemia/dehydration from osmotic diuresis, an increased risk of hypoglycemia when used with insulin or sulfonylureas, and the potential for euglycemic DKA (74).

While GLP-1 receptor agonists (GLP-1RAs) such as semaglutide offer significant metabolic benefits, their use in athletes is primarily complicated by gastrointestinal (GI) disturbances. Clinical trials have shown that subcutaneous semaglutide can cause nausea in up to 20% of patients and vomiting in up to 11.5%, while oral formulations may lead to GI disturbances in as many as 56% of cases (75). For athletes, these effects, including delayed gastric emptying, can significantly hinder nutritional intake and fueling during exercise. Additionally, while GLP-1RAs do not increase the risk of hypoglycemia when used with metformin, the likelihood of clinically significant hypoglycemic events increases when they are co-administered with sulfonylureas or insulin (76). Therefore, it is important to proactively reduce the dosage of secretagogues or insulin when initiating GLP-1RA treatment to ensure exercise safety. These challenges are summarized in Table 1.

**Table 1:** Medications for type 2 diabetes management and their challenges.

Medications	GI tolerance	Risk of hypoglycemia	Risk of dehydration	References
SGLT2 inhibitors (Empagliflozin, Dapagliflozin)	Neutral	Low in monotherapy	High (Monitor for syncope/orthostasis)	(74)
GLP1 receptor agonist (Semaglutide (brand name: Ozempic))	Poor	Contextual (increase with sulfonylureas)	Neutral	(75)

## 6 Nutritional requirements for athletes with diabetes

A healthy diet is essential for promoting physical activity, improving athletic performance, and speeding up recovery after exercise. It is expected of professional athletes to fully satisfy their nutritional needs by eating foods of the right kind and amount (77, 78).

Athlete’s dietary requirements are mostly determined by the demands of their activities and the objectives they set for themselves in order to achieve optimal health and sports performance. Following healthy eating habits is essential since it affects almost every body function, from energy production to recuperation after exercise. Moreover, education, attitude, and the accessibility of nutrition-related information sources can all have an impact on a person’s dietary decisions (79). A summary of macronutrients and their dose-timing-type is described in Table 2.

**Table 2:** Dose-timing-type of macronutrients adjusted for athletes.

Macronutrients	Dose (quantity)	Timing	Type (quality)	Safety caveats for athletes	References and grading
Carbohydrates	3–12 g/kg/day (Scaled to duration/intensity)	1–4 g/kg at 1–4 h pre-exercise; continuous during endurance	Low-GI/GL for basal meals; High-GI/Hydrogels during exercise	High-GI during exercise is a safety tool to prevent hypos and spare glycogen.	(77, 78) (Grade C); (91) (Grade A)
Protein	1.2–2.0 g/kg/day (1.5 g/kg avg. for endurance)	Distributed every 3–5 h; 3–4 h pre-exercise	High-quality/Amino acids for Skeletal Muscle & Connective Tissue	50 g post-exercise protein bolus increases GLP-1 and glucagon, reducing nocturnal hypo risk.	Gillen et al. (98) (Grade B); Muntis et al. (102) (Grade B)
Fat	20–35% of total caloric intake	Consume with/after meals; avoid high-fat immediately pre-race.	Omega-3 PUFAs (450–900 mg safe range); SFA < 10%	Diets high in SFA decrease insulin sensitivity via non-esterified fatty acid (NEFA) buildup.	Ghazzawi et al. (84, 85); Parry et al. (115) (Grade B)

### 6.1 Carbohydrate needs: timing, type and quantity

Performance during endurance and intensive training can be greatly improved by consuming a diet heavy in carbs. By increasing the accessibility of external carbohydrates and conserving them as glycogen in the muscles and liver, this is achieved (80). Endogenous carbohydrates are gradually depleted by energy expenditure during training. The length and intensity of exercise determine how much of this depletion occurs. As one of the macronutrients that may be metabolized both aerobically and anaerobically, carbohydrates are very important for athletic performance (81, 82). The two main energy sources for working muscles are muscle glycogen and blood glucose. It has been shown that maintaining a high level of intensity during exercise becomes more difficult as glycogen stores decline (83). Eating a high in carbohydrates appetizer or meal before working out results in the best muscular glycogen storage. However, insufficient pre-exercise glycogen causes protein degradation, glycogenolysis, muscle glycogen depletion, early tiredness, and lower training intensity (81, 84, 85). Reaching the ideal carbohydrate intake promotes recovery and maximizes glycogen storage for training sessions that follow.

To estimate their daily carbohydrate needs, athletes are recommended to ingest 3–12 g of carbohydrates per kg of body weight; the precise amount will vary depending on the duration and intensity of their physical activity (Grade C). Individual differences and the comfort of their gastrointestinal systems should also be considered (86).

It is generally advised to consume 1–4 g·kg − 1 body mass (BM) of CHO 1–4 h before engaging in endurance exercise (77, 78) (Grade C). The choice of pre-exercise meal is influenced by a number of variables, including sex, training status, and/or the dietary habits of endurance athletes (87); yet, CHO consumption before to exercise seems to be significant independent of personal preferences.

Many people will list items like potatoes, rice, and pasta as “typical” CHO sources when thinking about their pre-exercise CHO intake. In addition to ingesting it prior to an activity, it is believed that CHO consumption throughout endurance exercise is essential for maintaining productivity. During competition, endurance athletes usually favors transportable CHO supplements in the form of hydrogels, injections, bars, and chews due to their improved oxidation rates, gastrointestinal tolerability, and CHO absorption (88). However, consuming foods and fruits high in CHO may seem like a natural and affordable way for endurance athletes to get CHO while working out (89).

### 6.2 Glycemic index and glycemic load

It is highly advised to use medical nutrition therapy before starting pharmacological therapy since it provides an evidence-based method of managing diabetes through lifestyle changes. Low glycemic index and/or low glycemic load dietary patterns have been demonstrated to improve glycemic management in diabetic patients, and it is generally agreed that the quality and quantity of carbs are the primary determinants of glycemic response (227) (Grade C). A carbohydrate-rich food’s glycemic index (GI) estimates how fast the carbs are taken into the bloodstream and how quickly they break down after digestion.

Foods are categorized as low (GI ≤ 55), medium (GI 56–69), and high (GI ≥ 70) based on GI, and as low (GL ≤ 10), medium (GL 11–19), and high (GL ≥ 20) based on GL. The GI and GL categorization systems were created arbitrarily, meaning they have nothing to do with the food’s nutritional density or any risk factors for chronic diseases that could result from eating it (90).

Low-GI diets have been shown to lower HbA1c and improve the overall glycemic profile when used as an intervention for people with type 2 diabetes (91) (Grade A). However, postprandial hyperglycemia is still very common in people with T2D even when the overall glycemic profile seems to be good. Therefore, a low-GI/GL diet in general, without taking into account the kind and quantity of carbohydrates in each meal, may be an insufficient strategy when especially aiming to manage postprandial glucose variations. In order to minimize elevated spikes in the after meals state throughout the day, it is imperative to take into account the dietary content of each meal separately (92) (Grade C).

Low-GI diets typically contain more fibre, which has been demonstrated to enhance postprandial oscillations by instantly reducing glycemic response. Numerous earlier research has documented the positive impact of fibre, particularly soluble or viscous fibre, on postprandial glucose excursions (93) (Grade A).

### 6.3 Carbohydrate digestion and absorption

The degradation of carbohydrates in the mouth is started by salivary amylase. After being broken down in the digestive tract, monosaccharides are taken up by the bloodstream. The gut lining allows simple sugars like glucose, fructose, and galactose to be absorbed after complex carbs like starch are hydrolyzed by enzymes during digestion. The pancreas releases more insulin when blood sugar levels are raised by eating carbohydrates. Insulin directs the body’s cells to absorb glucose for energy or reserve. When blood glucose levels fall, the pancreas releases glucagon, which causes the liver to release stored glucose (94).

Glucose needs glucose cotransporters (SGLTs) and glucose transporters (GLUTs) to cross the bilayer lipid membrane and enter cells because of its hydrophilic nature (95). A member of the GLUTs family, GLUT2 has a comparatively high glucose transport activity and is primarily expressed on *β* cells and other tissues with high glucose concentrations, including the kidney, liver, intestine, and nervous system, all of which are vital for managing and responding to blood glucose metabolism. As a glucose sensor, GLUT2 carries glucose into cells in the physiological state and works with glucokinase (GCK) to quickly adjust the amounts of glucose on either side of the cell membrane to achieve equilibrium and match the concentration of glucose in the environment. After glucose enters the bloodstream, it is absorbed by pancreatic β-cells through GLUT2, which causes the release of insulin to preserve blood glucose homeostasis (96) (Table 3.).

**Table 3:** Nutritional consideration of diabetic athletes.

Nutritional consideration	Purpose/goal	Dietary strategy	Timing/implementation	Key notes for diabetic athletes	References
Carbohydrate Periodization	Match fuel to training demands	Adjust CHO intake based on training intensity	Pre-, intra-, and post-exercise	May prevents hypoglycemia and supports endurance	(200, 201)
Glycemic Index Awareness	Control postprandial glucose	Prefer low–moderate GI foods before exercise	1–3 h pre-exercise	May reduces rapid glucose spikes	(202)
Pre-Exercise Snack	Prevent hypoglycemia during exercise	15–30 g of complex carbs + protein	30–60 min before activity	Adjust insulin dose accordingly	(203)
Intra-Exercise Fueling	Maintain blood glucose during long sessions	Sports drinks, gels, or fruit (30–60 g carbs/h)	For >60 min exercise	Frequent glucose monitoring advised	(204)
Post-Exercise Recovery	Replenish glycogen and promote repair	1–1.2 g carbs/kg + 0.3 g protein/kg	Within 30 min post-exercise	Insulin sensitivity is elevated post-exercise	(205)
Protein Intake	Support muscle recovery	1.6–2.0 g/kg/day total	Evenly distributed across meals	Helps stabilize blood sugar	(206)
Fat Intake Quality	Optimize long-term metabolic health	Focus on MUFAs and omega-3 s	Throughout the day	Supports cardiovascular protection	(207)
Hydration & Electrolytes	Prevent dehydration and ketosis	Include water + electrolytes (Na, K, Mg)	Before, during, and after exercise	Hyperglycemia increases fluid loss	(208)
Continuous Glucose Monitoring (CGM)	Track glucose fluctuations	Use CGM devices	Throughout day	Helps fine-tune nutrition and insulin timing	(209)
Insulin Timing Adjustment	Avoid hypo/hyperglycemia during training	Reduce bolus insulin before exercise	30–60 min pre-exercise	Coordinate with dietitian and physician	(210)
Carbohydrate Counting	Align insulin to food intake	Track total carb content of meals	Each meal/snack	Essential for flexible insulin dosing	(211)
Micronutrient Optimization	Support energy metabolism	Ensure adequate Mg, Zn, Cr, and Vit D	Through balanced diet or supplements	Deficiencies may impair insulin sensitivity	(212)
Fiber Intake	Stabilize glucose absorption	Include whole grains, legumes, veggies	Each meal	May slows glucose rise and improves satiety	(213)
Avoiding Late-Night Hypoglycemia	Prevent nocturnal lows	Small bedtime snack (carbs + protein)	Before sleep	Adjust insulin accordingly	(214)
Caffeine Use	Enhance alertness/performance	Limit to 3–6 mg/kg	30–60 min before exercise	May increase glucose variability	(215)
Alcohol Management	Prevent delayed hypoglycemia	Pair with carb-containing foods	Occasional, post-training	Monitor glucose overnight	(211)
Ketone Monitoring	Detect potential diabetic ketoacidosis	Use urine or blood ketone tests	During illness or extreme exercise	Particularly vital for Type 1 diabetes	(216)
Meal Frequency	Maintain steady glucose levels	4–6 smaller balanced meals/day	Throughout day	Reduces large glucose fluctuations	(209)
Individualized Nutrition Plan	Personalize to metabolic response	Dietitian-guided plan	Continuous	Integrate training, insulin, and meal data	(213)

### 6.4 Protein requirements: muscle repair and glycemic control

The protein requirement, which is mostly dependent on whole-body assessments of protein metabolism, can be described as “the minimum daily protein intake necessary to satisfy the metabolic demands of the body which includes the maintenance of body composition.” The protein suggestion, on the other hand, is based mostly on tissue-specific (mostly skeletal muscle) assessments of muscle metabolism and can be described as “protein strategies to optimize performance in athletes by facilitating training adaptation and/or accelerating recovery” (97). The average protein consumption among endurance athletes participating in rowing, swimming, ice skating, road cycling, running, and ultra-endurance sports was approximately 1.5 g·kg^−1^·day^−1^. This was bolstered by total energy intakes of almost 12.3 MJ/day for men and 10 MJ/day for women (98).

All tissues are composed of amino acids, which are the building blocks of proteins. After intense training sessions or tournaments, athletes can restore their skeletal muscle and connective tissues with the support of protein in their diet. Protein is primarily consumed by athletes for the repair and restoration of both skeletal muscle and connective tissues after intense exercise or competition. The degree to which muscles reconstruct following training depends on the kind, quantity, and timing of protein consumption. It is important to stress that athletes do not primarily rely on protein as their fuel source (99).

Protein intake before, during, and after exercise is crucial for promoting muscle remodeling and repair as well as for boosting responses related to strength and hypertrophy. Positive effects on muscle protein synthesis (MPS) have been associated with protein intake throughout these periods (100). When combined with resistance exercise, eating protein 3–4 h before working out can assist maintain muscle growth and enhance muscle recovery (101).

According to the results of a study, higher daily protein consumption—1.2–2.0 g/kg/day is the recommended daily intake level in sports nutrition—may enhance the post-exercise glycemic response, particularly in people who receive various daily insulin injections for the treatment of their diabetes, those who are overweight or obese, and female adolescents (102) (Grade B). Although there is not much research on how dietary protein intake affects exercise-related glycemia in individuals with type 1 diabetes, a recent laboratory-based pilot study discovered that a 50 g protein bolus after moderate-intensity exercise raised levels of glucagon, glucagon-like peptide-1 (GLP-1), and gastric inhibitory peptide (GIP) overnight when compared to water after exercise. This resulted in a lower need for glucose infusion to maintain euglycemia (103).

Additionally, it has been proved that high-protein diets enhance insulin sensitivity in individuals with type 2 diabetes by decreasing intra-hepatic liver triglycerides and raising post-meal glucagon secretion (104, 105). Improvements in post-exercise glycemia could be attributed to decreased stomach emptying rates and increased insulin sensitivity brought on by higher protein consumption.

### 6.5 Mechanism

#### 6.5.1 Protein synthesis and breakdown

**Figure 1:**
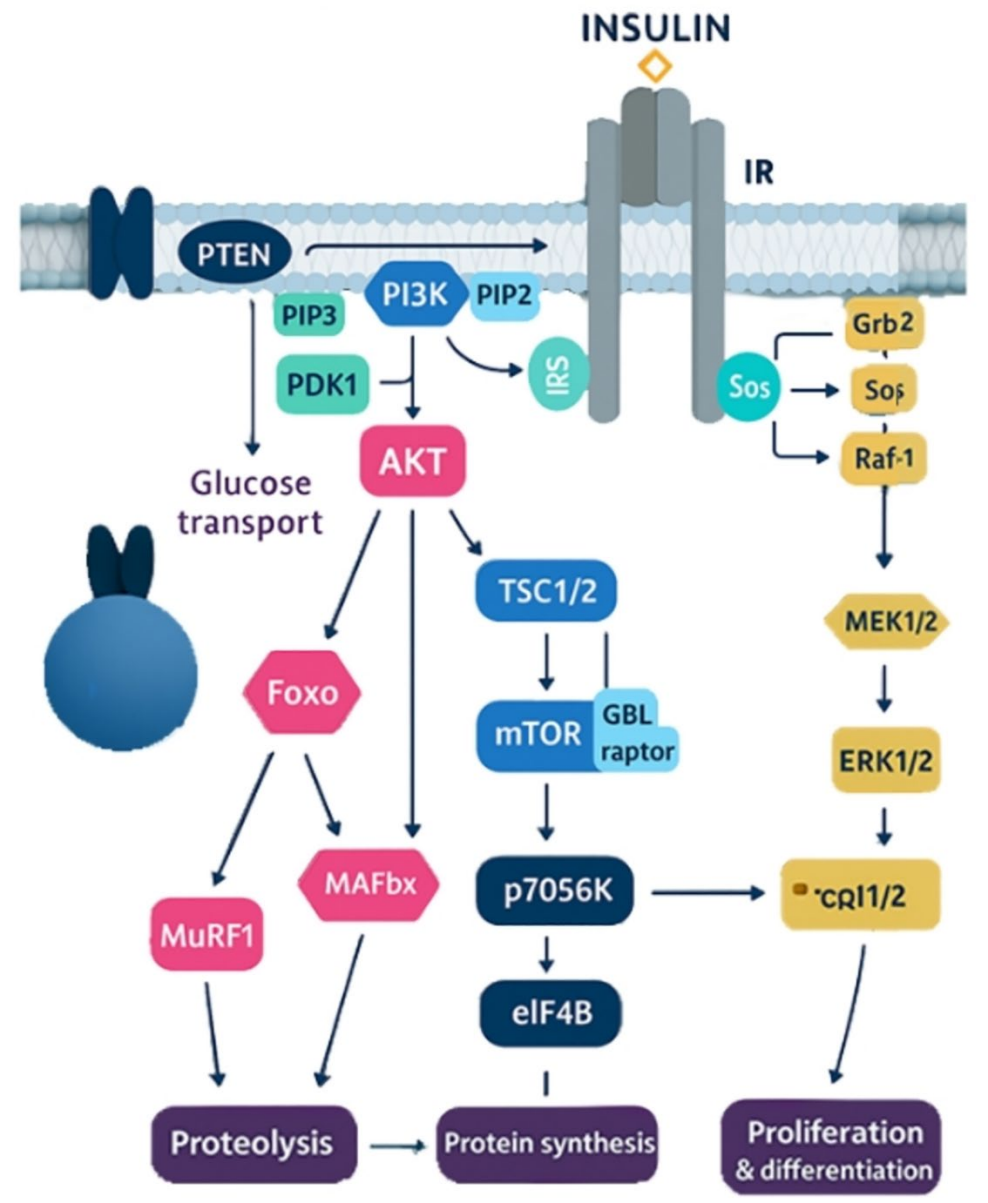
Impact of dietary protein on glycemic control.

The overall balance between muscle protein synthesis (MPS) and breakdown (MPB) separates the anabolic (synthesis exceeds breakdown) and catabolic (breakdown exceeds synthesis) periods. Since nonessential amino acids are normally readily available in muscle, the anabolic response is regulated by the intracellular availability of essential amino acids (EAA), which are generated from MPB or inward influx from plasma (Figure 1).

Intracellular EAA may be charged to the proper transfer ribonucleic acid (tRNA) for protein synthesis, oxidized, or efflux back into plasma. The elevated rate of MPB, the primary cause of intracellular EAA emergence in the following absorptive state, largely dictates the quantity of intracellular EAA available as antecedents for MPS. In the post-absorptive condition, MPB will always be greater than MPS due to the imperfect efficiency of intracellular amino acid recycling, which causes a net loss of muscle protein. Exogenous EAA are necessary to raise circulation concentrations to stimulate MPS and decrease MPB in order to restore the lost muscle protein (106). Therefore, the main factors causing an increase in MPS and the accompanying growth of the skeletal muscle protein pool are dietary EAA. Skeletal muscle is essential for function, but it also stores EAA for splanchnic organs and tissue in times of stress or inadequate nutrition (107).

#### 6.5.2 Amino acid metabolism and insulin secretion

The most popular explanation for the improved glycemic control seen with high protein diets (HPD) is the amino acids’ purported insulinotropic action (108). Amino acids can accomplish this by directly influencing the function of pancreatic *β* cells and, to a lesser extent, *α*-cells. This could be explained by a number of methods. The tricarboxylic acid cycle, which needs oxidized amino acid substrates to produce adenosine triphosphate (ATP), is one of the processes that make up the triggering route. The β-cell membrane depolarizes as a result of ATP-sensitive potassium channels closing. β-cell depolarization is enhanced by cationic amino acids like arginine and lysine. Additionally, it is believed that β-cell depolarization is facilitated by the co-transportation of proline and l-alanine with sodium ions (109). Calcium channel activation brought on by depolarization causes intracellular calcium influx, which in turn causes insulin exocytosis (110). Glutamate may regulate calcium-induced insulin exocytosis, according to evidence.

Another important factor in amino acid-mediated insulin production is the function of the protein kinase signaling complex known as the mammalian target of rapamycin complex 1 (mTORC1). By coordinating downstream mRNA translation and enhancing the ribosomal machinery’s capability, mTOR controls protein synthesis, particularly insulin manufacturing in the β cell (109).

Glycemic management and recovery are supported by adequate protein intake because it increases insulin release, improves muscle uptake of amino acids, and facilitates effective glycogen and protein resynthesize.

### 6.6 Fat intake: energy source and insulin sensitivity

Athletes must consume the right quantity of energy throughout both activity and recovery in order to perform at their best. Whereas carbohydrate catabolism can happen with or without oxygen, fat oxidation is largely dependent on it. Notably, more ATP is produced when glucose is completely broken down in the mitochondria with oxygen present (111). It is crucial to consume enough fat, but diets that are too heavy in fat or that load the body with fat do not work (84, 85). For athletes, dietary fat should make up 20–35% of total caloric intake, with <10% coming from saturated fat.

One form of polyunsaturated fatty acid (PUFA), omega-3, serves as a structural element in phospholipid cell membranes. Omega-3 is essential for the body’s inflammatory response (112). Omega-3 has been associated with improved anaerobic endurance capacity, delayed onset muscle soreness, increased oxygen efficiency during aerobic exercise, support for skeletal muscle health, and reduction of oxidative stress brought on by exercise in athletes. For best absorption, omega-3 should be taken with or after a high-fat meal (113).

**Figure 2:**
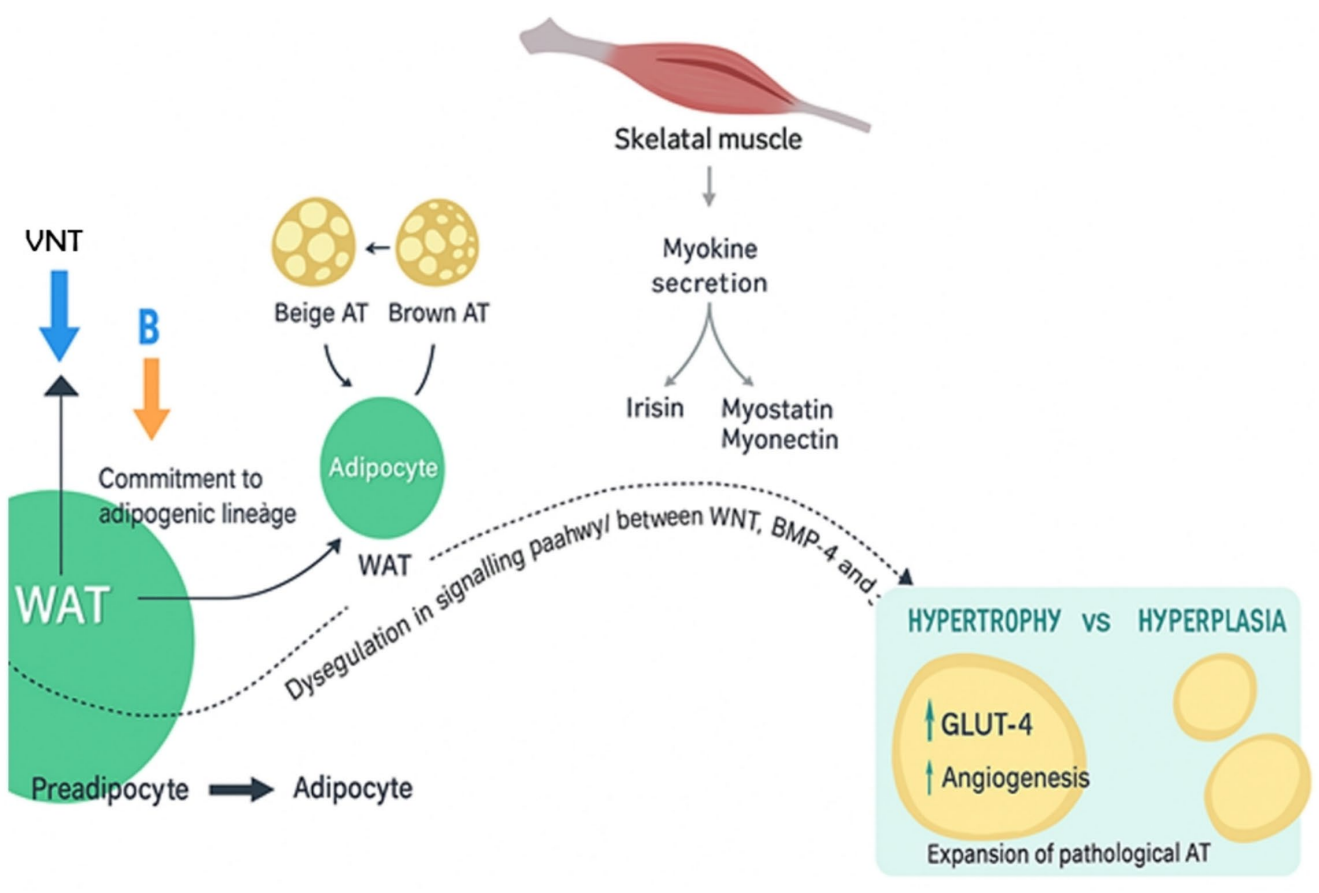
Insulin resistance in adipose tissue.

Insulin resistance has been linked to diets high in fat or diets enriched with saturated fatty acids (SFAs) (114). According to study findings, eating a diet high in SFA for 3 months decreased insulin sensitivity in healthy adults compared to eating a diet high in monounsaturated fatty acids (MUFA). It is yet unknown how a high total fat or SFA diet contributes to insulin resistance. According to one theory, insulin resistance results from the buildup of non-esterified fatty acids (NEFA) in non-adipose tissue organs including the liver and skeletal muscle caused by adipose tissue malfunction, which causes an excess of NEFA in the blood (115) (Figure 2).

### 6.7 Mechanism

#### 6.7.1 Fatty acid oxidation and insulin resistance

Insulin resistance has been linked, at a molecular level, to ectopic lipid deposition in non-adipose tissues and chronic low grade systemic inflammation. In fact, according to the “lipotoxicity theory,” insulin resistance development in peripheral metabolic organs, specifically the liver and skeletal muscle, is caused by cytosolic ectopic accumulation of fatty acid metabolites, such as ceramides and/or diacylglycerols (DAG). Conversely, the “inflammatory theory” proposed that insulin resistance is associated with increased tissue and systemic inflammation. The insulin receptor/insulin receptor substrates 1 (IRS1) axis is inhibited by stress-sensitive Ser/Thr kinases like Jun kinase (JNK) and IkB kinase (IKKβ), which are hyper activated as a result of inflammation brought on by a high-fat diet (116).

As a result, diets heavy in saturated fat or excessive calorie intake may encourage these molecular alterations, underscoring the significance of fat quality for athletes’ metabolic health.

#### 6.7.2 Effects on inflammation and oxidative stress

Numerous metabolic pathways are involved in the etiopathogenesis of insulin resistance; however, in recent years, it appears that all of these pathways are either directly or indirectly related to elevated inflammation. As was previously mentioned, it has been proposed that an increase in lipid metabolites such as ceramides and DAG activates kinases that impede insulin signaling, primarily at the IRS1 level (116). By encouraging oxidative stress and mitochondrial dysfunction, high-fat diets worsen these molecular abnormalities and further impede insulin signaling.

A study revealed that oxidative stress, hepatic lipid deposition, insulin resistance, and mitochondrial dysfunction are all present in high fat-induced obese mice (117). By causing oxidative stress and mitochondrial problems in kidney cells, long-term high-fat diet feeding encourages excessive apoptosis in mouse HK-2 cells (118). These results emphasize how crucial dietary fat quality is for preserving insulin sensitivity and metabolic health, especially in athletes or those at risk for metabolic diseases.

#### 6.7.3 Micronutrient considerations: vitamins, minerals and antioxidant

Micronutrients, which include vitamins and minerals that promote development, reproductive activities, and general wellbeing, are essential for maintaining life. Since the human body is not capable to make these necessary components, which are needed in minute amounts, they must be supplied from diet. Vitamins are classified according to how soluble they are; B and C are water-soluble, while A, D, E, and K are fat-soluble. Conversely, minerals are inorganic substances that support physiological processes. From the standpoint of an athlete, several essential vitamins and minerals are necessary for adaptation and peak performance (119).

#### 6.7.4 Zinc: a trace mineral

It is interesting to note that, despite their higher dietary intake, athletes frequently have reduced blood zinc concentrations than the general population, according to a prior comprehensive analysis (120). This implies that athletes’ zinc needs are probably higher than those of the general public, perhaps as a result of sweat losses brought on by prolonged, rigorous exercise (Table 4).

**Table 4:** Micronutrient and supplement considerations for diabetic athletes.

Nutrient/supplement	Physiological role	Potential benefits in diabetes	Interactions	Food sources	References
Magnesium	Cofactor in glucose metabolism	Improves insulin sensitivity	Excess can cause diarrhea	Leafy greens, almonds	(217)
Zinc	Antioxidant and insulin synthesis	Enhances wound healing, immune function	High doses may interfere with copper	Pumpkin seeds, meat	(218)
Chromium	Insulin cofactor	Improves glucose tolerance	Excess may cause renal stress	Broccoli, whole grains	(219)
Probiotics	Gut microbiota balance	Improves insulin sensitivity and immunity	Choose clinically tested strains	Yogurt, kefir, fermented foods	(220)
Omega-3 (Fish Oil)	Anti-inflammatory	Reduces triglycerides, improves endothelial function	Monitor for bleeding risk	Salmon, cod liver oil	(221)
L-Carnitine	Fat metabolism	Enhances endurance and recovery	Possible mild GI discomfort	Red meat, supplements	(222)
Alpha-Lipoic Acid	Antioxidant and glucose metabolism	Improves neuropathy symptoms	Monitor for hypoglycemia	Spinach, broccoli	(223)
Electrolytes (Na, K, Mg)	Fluid and nerve balance	Prevents dehydration and cramps	Adjust for sweat losses	Coconut water, electrolyte mix	(224)

#### 6.7.5 Other micronutrients

Iron, folic acid, and vitamin B12 are important factors in the development of red blood cells from a haematological standpoint (121). Iron’s significance is well known due to its essential function in the synthesis of enzymes essential to the electron transport chain at the cellular level and haemoglobin, which is integrated into red blood cells. Iron is therefore essential for vital functions including energy synthesis and oxygen transfer, both of which are very important for athletes (122). However, it is estimated that approximately 10% of male athletes and 35% of female athletes suffer from iron deficiency.

B vitamins are known to play a major role in haematological function in active people, in addition to iron. Of the nine B vitamins that are present in the diet, cobalamin (B12) and folate (B9) are essential for promoting the bone marrow’s ability to produce red blood cells (123).

## 7 Clinical optimization strategies: a multidisciplinary approach for doctors, athletes, and caregivers

The large degree of intra-individual and inter-individual differences in glycemia during a particular exercise session is one of the primary obstacles to the clinical management of exercise in T1DM.

Role of Physician: Athletes or their carers need to be informed by a doctor that while a particular exercise mode can be somewhat replicated in a fasted state with basal insulin conditions (124, 125), consistency is lost when the activity is performed in a fed or post-absorptive state (126), or when high glucose levels occur during a sporting event (127). It is believed that a number of factors contribute to the variation in glucose responses to exercise both within and between people with type 1 diabetes and a physician must help patient to account for these factors. Exercise mode, duration, and intensity are among these factors, along with a number of other event-level and participant-level attributes like time of day, exercise type, timing and amount of insulin administered, foods consumed, fitness level, sex, sleep status, stress level, individual insulin sensitivity, baseline glycemic control, and pre-exercise glucose concentration.

One of the most crucial therapeutic ideas that doctors should teach patients with type 1 diabetes who want to exercise is the phenomenon of Insulin On Board (IOB). It also limits spontaneous exercise, which may discourage people with T1DM from exercising but a physician provides rescue strategies. To help keep blood glucose from falling, one potential tactic is to take a little (mini) dosage of glucagon right before activity. This strategy helps prevent rapid blood glusice decline during activity (128). IOB in circulation is influenced by both basal and prandial insulin. While most devices only report prandial (i.e., bolus) insulin as IOB, people with type 1 diabetes who are using any kind of continuous subcutaneous insulin infusion (CSII) device can see how much insulin the pump calculates is “on board.” When insulin basal rate reductions are started approximately 90–120 min prior to the start of endurance activity (129), especially in the morning before the first meal (130), significant hypoglycemia protection is achieved for individuals on standard CSII medication.

**Figure 3:**
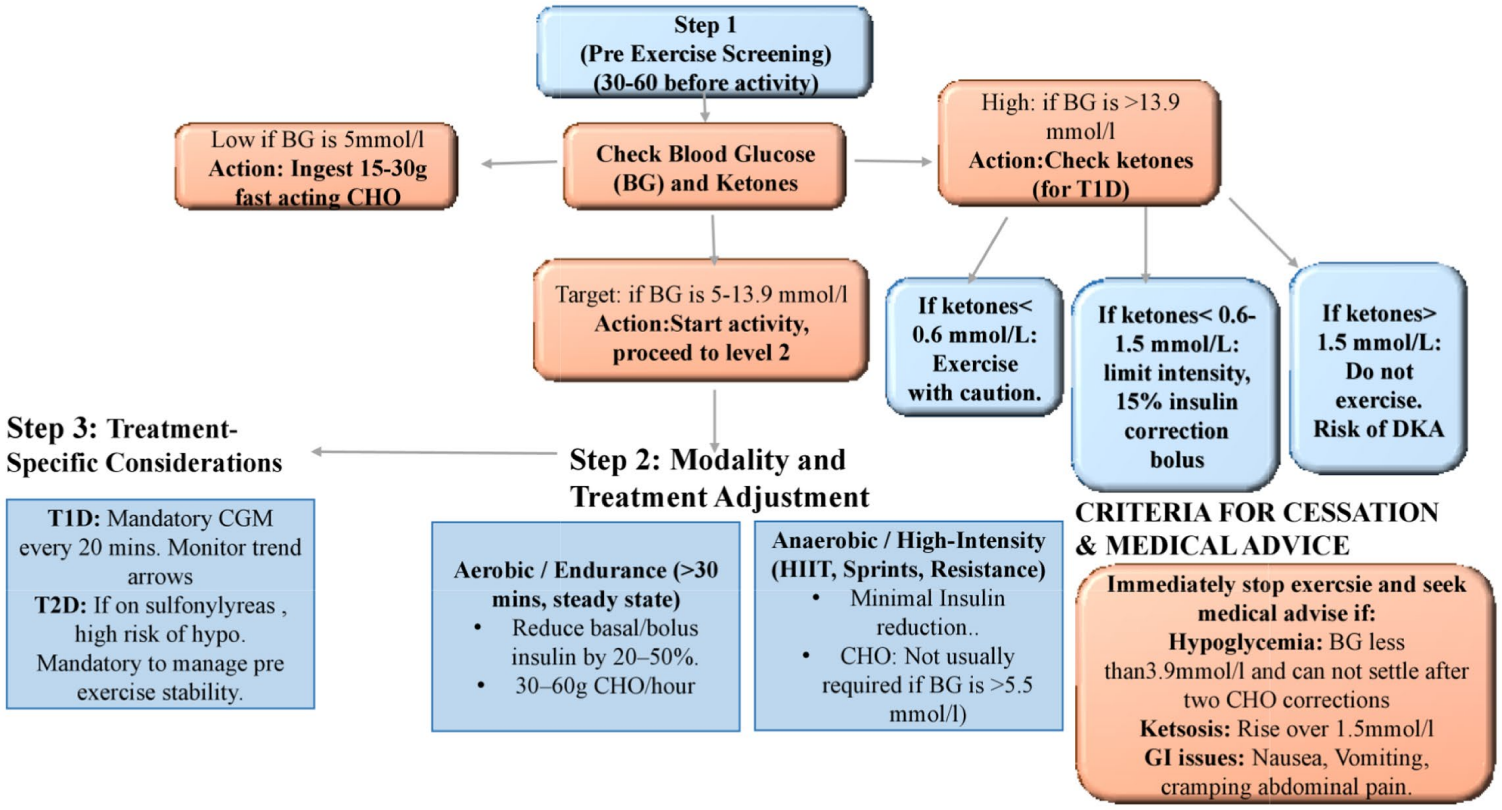
Practical applications and decision support.

The following algorithm shows a decision tree for management of Exercise induced Excrsuions among athletes with diabetes (Figure 3).

*Practical considerations for caregiver*

While the physician provides clinical guidance, caregiver’s provide practical hands-on-optimization during active play.

*Infusion site integrity:* For athletes who participate in contact sports, carers must make sure that infusion sites are regularly changed and rotated to guarantee proper insulin delivery with AID devices (131).

*Managing sensor accuracy:* Managing the CGM lag time during abrupt glucose increases and making sure that sensor callibrations are prevented during exercise are crucial responsibilities of the carer. Athletes who wish to calibrate should do so during a time that typically has minimal IOB, no food or exercise altering glycemia, and a flat trend arrow (e.g., first thing in the morning), as many CGM systems are currently factory-calibrated and do not require human calibrations.

*Fingerstick rule:* Performing a fingerstick blood glucose measurement and being ready to supplement with carbs early while athletes are exercising and see downward glycemic trend arrows or feel symptomatic symptoms to hypoglycemia is another tactic that a carer might assist with (132).

## 8 Glycemic control strategies for athletes with diabetes

For athletes with diabetes, maintaining ideal glycemic control is crucial to enhancing performance, avoiding hypoglycemia, and advancing general health. Individual insulin and glucose requirements, as well as the timing, intensity, and duration of exercise, must all be taken into consideration while developing strategies. Careful preparation prior to, during, and following exercise is necessary for effective management. This includes dietary, pharmaceutical, and monitoring changes.

### 8.1 Detailed workflow for glycemic management among athletes

Pre- exercise (90–120 before activity).

*The objective:* Enhance performance by maximizing muscle glycogen saturation (133).

*The loading protocol:* Target a carbohydrate intake of 8–12 g/kg/day; it is important to note that athletes frequently do not achieve this goal (134).

*The timing:* Emphasize the 60-min period before exercise to enhance performance (135).

*Insulin management:* Modify insulin doses to accommodate exercise-induced changes in insulin sensitivity (136).

### 8.2 Mechanism

#### 8.2.1 Molecular mechanism of glycogen supercompensation

Commonly found in cell cytosol, glycogen is a fuel source that makes up 2% of the volume of cardiac cells, 1–2% of skeletal muscle cells, and 5–6% of liver cells. Liver cells can have glycogen particles that are 10 times larger than those seen in skeletal muscle cells, and each one contains more than 50,000 glucose molecules (137). Weight gain is a notable reaction to glycogen super-compensation in many athletes because each gram of glycogen is stored with at least 3 gram of water (138).

### 8.3 During exercise

*Fueling strategy:* Consume 30–60 g of carbohydrates (CHO) per hour for sessions lasting more than 60 min. Inadequate intake can lead to reduced calcium release and increased fatigue (139).

*Safety monitoring:* Check blood glucose (BG) levels every 30 min (2).

*Intensity progression:* Gradually increase workout intensity to avoid sudden spikes. For cardiovascular activities, start by adding 5–10 min to the duration before increasing speed (2).

*The 90-minute rule:* If exercising within 90 min of insulin administration, adjust the dosage before starting to prevent excessive reduction in BG levels (2).

### 8.4 Mechanism

#### 8.4.1 Glucose uptake and utilization during exercise

According to earlier research, aerobic exercise is an effective approach for enhancing inflammatory markers (140) and glucose metabolism (141) in sedentary persons. Through improved oxygen utilization, aerobic training contributes significantly to body fat reduction. According to earlier research, aerobic exercise enhances muscular capillarity, which facilitates the diffusion of glucose from capillaries into muscle cells (142). It also alters the type of muscle fibre, flavouring substrate oxidation, and increases the number and size of mitochondria. Additionally, aerobic exercise raises lipid turnover and/or oxidation1 and the expression and/or activity of important signaling proteins that control glucose uptake and metabolism in skeletal muscle, such as GLUT4 translocation controlled by AMPK (143).

Skeletal muscle hypertrophy, which raises the mass of tissue insulin-sensitive in terms of glucose storage and utilization, may be partly responsible for the positive impact of resistance exercise on glucose metabolism outcomes. Additionally, resistance exercise may improve insulin action by promoting the development and function of insulin receptors, glucose transporters, and other glucose metabolism-related enzymes (142).

### 8.5 Post exercise recovery

Post-exercise management involves the need for rapid glycogen resynthesis and the management of increased insulin sensitivity to prevent nocturnal hypoglycemia.

#### 8.5.1 Recovery phase

*The “Golden Window”:* Muscle glycogen restoration is most efficient in the first two hours through an insulin-independent pathway (144).

*Timing Action:* Consume carbohydrates immediately. Delaying intake by just 2 h results in significantly lower glycogen concentrations and reduced performance the following day (145).

*Nutritional type:* Opt for a Maltodextrin-Fructose mix in a 1.5:1 ratio. This combination can enhance endurance capacity recovery by approximately 33% compared to glucose alone (146).

#### 8.5.2 The saturation phase (24–36 h post-exercise)

*Glycogen target:* In order to fully replenish muscle glycogen reserves, athletes should aim for a carbohydrate intake of 7–12 g/kg/day (81) (Grade C).

*Liver recovery:* It is important to recognize that liver glycogen resynthesis occurs at a slower rate, usually reaching saturation between 11 and 25 h after exercise (147).

Exercise-induced insulin sensitivity persists into the subsequent day (148) (Table 5).

**Table 5:** Comparison of insulin delivery technologies (225).

Features	MDI (multiple daily injections)	CSII (continuous subcutaneous infusion insulin pump)	AID (automated insulin delivery)
Mechansim	Basal-bolus therapy, administering insulin through subcutaneous injections to replicate the body’s basal and mealtime insulin needs.	Continuous subcutaneous infusion delivers insulin continuously to mimic the function of the pancreas by providing both basal and bolus insulin	CGM-integrated algorithms enable predictive auto-adjustment of insulin doses based on continuous glucose monitoring data.
Clinical Pros	Minimal maintenance; Easy to use with no issues of “on-body” equipment during contact sports.	Reduced risk of hypoglycemia and eliminates the need for multiple daily injections, ensuring high dosing precision.	Offers the highest level of safety by predicting and suspending insulin before hypoglycemia occurs.
Clinical Cons	Fixed kinetics make it challenging to reverse the effects of insulin once it has been injected, increasing the risk of improper dosing	Insulin pumps come with the risk of malfunction or site occlusion, as well as potential skin problems and can be bulky during physical activity	Continuous glucose monitoring (CGM) is essential for insulin pump users, but it requires consistent wear and comes with a higher cost. There is also a potential for “rebound” hyperglycemia when using insulin pumps.
Efficacy	Comparable glycemic effectiveness to CSII in various settings	It may achieve a higher proportion of Time Below Range (TBR) targets.	It is superior in preventing nocturnal hypoglycemia after exercise.
Exercise Strategy	Reduce the rapid-acting bolus dose by 25–75% for the meal consumed 1–3 h prior to exercise.	Initiate a temporary basal rate reduction of 50–80% 60–90 min before starting the activity.	Activate “Activity Mode” 1–2 h before exercise to facilitate a decrease in insulin levels.

## 9 Performance optimization for athletes with diabetes

In the ever-changing realm of sports, striving for perfection and optimizing athletic performance are timeless undertakings. Every level of athlete, from inexperienced hobbyists to seasoned pros, is always looking for methods to improve their general wellbeing, mental toughness, and physical prowess. Athletes can realize their full potential and succeed in their respective sports over the long term by taking into account the interaction of training, diet, and recuperation.

### 9.1 Carbohydrates periodization: manipulating intake for performance

Periodized CHO availability is a hybrid strategy to training nutrition that, depending on the athlete’s performance objectives and training needs, alternates periods of high-CHO (HCHO) consumption with sessions conducted under low-CHO availability (149). Instead of replacing a high-fat diet over time, these “train low” tactics are used abruptly and sporadically (for one or two sessions at a time), purposefully limiting carbohydrate consumption for a brief period of time (150).

Athletes must have adequate energy availability from carbs in order to meet the demands of competition and recuperate for future events (151). The ability to do high-intensity exercises depends on the induction of glucose metabolic pathways. Adenosine triphosphate (ATP) synthesis can be powered by both lipids and carbohydrates, however during high activity intensities, carbs are primarily utilized. According to research, using carbohydrates rather than fats is necessary to sustain the levels of activity found in elite athletes (152).

Since the depletion of glycogen is a performance limiting issue, athletes should begin bouts with full glycogen reserves (149). It is currently advised to consume 1–4 g per kg BW of CHO proportionately 1–4 h prior to high-intensity activity.

Sports drinks with 1.5 g of CHO per 25 mL of water can also be used as a mouthwash during a game (5 to 10 s every 10 min) to provide an extra source of carbs for athletes who prefer not to consume sports drinks directly. CHO solutions (such as glucose, sucrose, or maltodextrin) were evaluated during games at concentrations ranging from 6 to 12%, and it was observed that the running time and time to tiredness improved (153). Within the first 4 h following a game, a post-exercise CHO intake of 1 to 1.5 g per kg BW per hour may promote maximal glycogen resynthesize (154).

### 9.2 Mechanism

#### 9.2.1 Adaptations to different carbohydrates diets

Current sports nutrition guidelines state that high carbohydrate (CHO) availability (77, 78), which is defined as balancing the body’s limited CHO storage with event-specific fuel requirements for the muscles and central nervous system is the best way to maintain prolonged high-intensity endurance sports performance (155).

The renewed interest in adapting to low-CHO, high-fat (LCHF) diets is a purposeful contrast to these recommendations, as it has been noted that athletes can significantly increase their already improved capacity for fat oxidation during exercise, including the exercise intensity at which maximal rates of fat oxidation occur (156). Both the precise multi-system physiological reactions to keto adaptation and the time frames needed to attain them are controversial (157). Nonetheless, the main modifications in relation to exercise performance seem to be enhanced absorption, delivery, and subsequent oxidation of free fatty acids.

Even in highly skilled endurance athletes who develop this trait of a high capacity for fat oxidation during exercise as a result of their training, there is unmistakable proof from both intervention (158) and cross-sectional studies (159) that following a ketogenic LCHF diet results in significant (e.g., 200–250%) increases in capacity for fat oxidation during exercise.

#### 9.2.2 Impact on performance and glycemic control

Even in endurance athletes whose training promotes such adaptations, low CHO, high fat (LCHF) diets increase the release, transport, absorption, and utilization of fat in the muscle tissue (160).

Even though training increases muscle oxidative capacity and fat utilization, adaptation to the ketogenic-low carbohydrate high-fat (K-LCHF) diet is accompanied by a significant increase in muscle fat oxidation capacity during exercise. The mechanism or mechanisms behind the improvement in fat utilization have not yet been directly examined in research involving K-LCHF and endurance sports. However, prior research utilizing non keto- low carbohydrate high fat (NK-LCHF) diets has documented increases in hormone-sensitive lipase, intramuscular triglycerides, and the expression of carnitine palmitoyl transferase and the fatty acid translocase FAT/CD36 protein. All of these alterations point to increased fat availability, mobilization, and transport processes under the intricate control of muscle fat utilization (156).

With pre-event CHO loading to maximize muscle glycogen content and/or CHO intake during the event to maintain high CHO availability for longer duration competitions, nutrition guidelines for endurance sports have been centered on methods to match the body’s limited CHO stores (155) to the event’s fuel costs for the past 60 years. When these methods maintain high levels of CHO oxidation throughout exercise and aid in motor recruitment, pacing, and effort perception, they improve endurance performance (161). In both lab and field settings, there have been observable improvements in the performance of professional athletes (133).

### 9.3 Caffeine and other ergogenic aids: safety and efficacy

Pharmaceuticals used to improve athletic performance are known as “ergogenic aids” (162). While athletes of all skill levels frequently use nutritional supplements and ergogenic aids, only a small number of these substances like creatine, sodium bicarbonate, and caffeine have been shown to improve athletic performance. Ergogenic aids with their primary mechanisms, sport context, Evidence strength and doping/ safety cautions are presented in Table 6. Health and sports officials are concerned about the increasing number of athletes utilizing ergogenic aids and nutritional supplements. It has been discovered that many of these aids and supplements are tainted with dangerous or prohibited ingredients. Should the products include banned doping substances, such contamination could endanger athlete’s health or result in their expulsion from competition (163).

**Table 6:** Clinical impact of ergogenic aids and targeted nutrients.

Ergogenic aid	Primary mechanism	Sport context	Clinical/glycemic impact	Evidence strength (ISSN)	Context of use	Doping
Caffeine	Adenosien receptor antagonsim, increases Adrenaline	Endurance and HIIT	Increases Blood glucose but may lower insulin sensitivity acutely.	Strong (Level 1) (176)	30–60 pre exercise	WADA monitored; may mask hypo symptoms.
Creatine	Increase in intramsucular creatine	Explosive	Decrease in blood glusose: Enhances non-insulin-mediated uptake.	Strong (Level 1) (6)	Daily loading (3-5 g)	Use NSF-certified; monitor renal health.
Beta-Alanine	Raises carnosine levels, Buffers intamuscular PH	HIIT	Neutral: Improves Buffering capacity	Strong (Level 1) (6)	Daily Loading (4–6 grams)	May cause skin tingling
BCAA’s	Leucine -Driven Muscle protein synthesis	All types	Mixed: May help in recovery but requires insulin for uptake.	Mixed (Level 2) (6)	Pre- workout	Potential for glycemic variability in fasted states.

### 9.4 Mechanism

#### 9.4.1 Caffeine’s effect on glucose metabolism and performance

A natural stimulant, caffeine is linked to a number of hypothesized ergogenic effects. Caffeine, which is well-known for its stimulating qualities, raises energy expenditure, stimulates neurotransmitter release, and improves cognitive function. Caffeine is a powerful ergogenic aid for both aerobic and anaerobic training, according to studies, and it especially helps with endurance exercises like cycling and running (164). According to research, taking 2–5 mg/kg of caffeine before to performance-based activities can greatly improve athletic performance (165).

In healthy individuals, acute caffeine consumption lowers insulin sensitivity, according to a new meta-analysis (166). Because the A1 and A2 subtypes of the adenosine receptor, which are involved in glucose absorption in skeletal muscles, are antagonistic to one another, acute caffeine treatment reduces insulin resistance and glucose tolerance. Additionally, the primary neurotransmitters of the sympathetic nervous system, noradrenalin and adrenalin, interact synergistically with coffee (167). Caffeine’s detrimental effects on glucose metabolism may be the cause of the acute consequences of coffee ingestion. Caffeine is likely the only element causing the acute effects because it is quickly metabolized in most people. Caffeine’s effects on glucose metabolism are in line with short-term trial results and at odds with epidemiological findings, which indicate that regular coffee drinking lowers the risk of type 2 diabetes (168).

The effects of caffeine supplements on endurance, muscular contraction, and perceived exertion are the main topics of several hypothesized mechanisms that attempt to explain how they affect athletic performance (165). By competing with adenosine for its receptors in the central nervous system, caffeine counteracts the negative effects of adenosine on neurotransmission, arousal, and pain perception. This is the main mechanism (86).

Caffeine’s analgesic effects also lessen the impression of pain and effort during exercise, which may be another route, particularly in uncomfortable workouts. As a result, decreased pain perception could help sustain or raise motor unit firing rates, which would enable the creation of more force (169).

#### 9.4.2 Other supplements: mechanisms and evidence

##### 9.4.2.1 Beta-alanine

When taken as a supplement, beta-alanine, a naturally occurring non-essential amino acid, raises muscle carnosine concentrations (170). By raising muscle carnosine levels, beta-alanine supplementation has been shown to be effective in improving performance during brief, high-intensity exercises. Strong evidence of its beneficial effects on a range of athletic metrics is provided by the systematic review and particular investigations. The suggested dosage plan emphasizes how important the first loading phase and the subsequent maintenance dose are for the best outcomes. Supplementing with beta-alanine may be an option for athletes and those who engage in high-intensity activities looking to improve their performance (171).

##### 9.4.2.2 Creatine: an ergogenic aid

The most popular and scientifically supported ergogenic aid is creatine (172). It is recommended over other ergogenic aids because of its demonstrated capacity to boost muscular strength, power, and fat-free mass, all of which improve exercise and athletic performance (173). In short-duration, maximal-intensity activities, research on creatine supplements consistently demonstrate improved performance and strength, as demonstrated by gains in metrics like single-repetition maximum, muscle strength, repetitions, muscular endurance, speed, and overall strength (6).

According to recent studies, creatine supplementation causes a rapid increase in intramuscular creatine, which has ergogenic benefits. Doses range from 0.3 g per kg per day for 3 to 5 days or 20 g per day for 5 to 7 days without interruption. Furthermore, it has been demonstrated that creatine supplements hasten the healing process following muscular damage and injury (174).

##### 9.4.2.3 Branched-chain amino acids

Combining three of the nine essential amino acids is the best technique to characterize branched-chain amino acids (BCAAs). The three BCAAs valine, leucine, and isoleucine cannot be produced by the body alone. Leucine stands out among the three BCAAs for its critical function in controlling muscle protein synthesis (MPS) and its modulatory effects, even when hyper aminoacidemia is present (175).

Furthermore, BCAA supplements work in a number of ways, including as lessening pain and halting the breakdown of muscle tissue during resistance and vigorous training. They enhance the MPS response, encourage muscular function recovery, and lessen central weariness (86).

Following the guidelines set forth by the International Society of Sports Nutrition (ISSN) Position Stands (176), this section focuses on supplements that are particularly important for managing blood sugar levels, maintaining hydration, and ensuring the safety of exercise in athletes with diabetes.

## 10 Classification of exercise classes and glycemic impact

Any workout session or event can generally be classified as either mixed, anaerobic, or primarily aerobic. These phrases, however, can be a little deceptive because any event involves multiple distinct energy systems, such as the phosphagens pathway, glycolysis, and oxidative phosphorylation, some of which require oxygen supply while others do not. In actuality, most types of exercise involve the simultaneous activation of numerous energy-generating pathways, with some pathways dominating during specific stages of the exercise session, such as at the beginning of the event or during near maximal effort, and other pathways dominating during later stages of the activity or when the intensity drops (177). Major Classes of sports (exercise) with their respective glycemic trend are shown in the Table 7.

**Table 7:** Classes of sports with their glycemic impact, CHO strategy and insulin adjustment (226).

Activity class	Primary energy system	Representative sports	Glycemic trend and risk	CHO strategy/grading	Insulin adjustment/ grading
Endurance	Oxidative Phosphorylation	Marathon, Distance Cycling	Decrease (High risk of hypoglycemia)	Short (<30 m): 10–20 g. Long (>1 h): 75–90 g/h. Ultra (>2 h): 90–144 g/h (Grade C)	25–75% reduction in bolus/basal if exercise is 2–3 h post-meal. (Grade B)
High Intensity Interval Trainning	Mixed (Glycolysis and Oxidative)	Running, Rowing, Cycling (10 s to 5 min)	Stable but can fluctuate	Fasted: 10–15 g. Post-meal (Hyperinsulinemia): 30–60 g/h. (Grade B)	Avoid evening exercise to prevent nocturnal hypoglycemia; monitor BG before sleep. (Grade C)
Explosive	Glysolsis and phosphagens	Sprinting, plyometrics (jumping), squats, agility drills and heavy weightlifting (10–20 s)	Rise in Glycemia	Pre-exercise: Minimal needed. Recovery: 1–1.2 g/kg/h within first 4 h. (Grade C)	Correction bolus may be needed for spikes, but adds risk of late-onset hypoglycemia

Since an individual’s energy expenditure is correlated with their age, size, and other factors, metabolic equivalents (METs) are frequently used to describe a person’s relative exercise intensity during activity. In individuals with type 1 diabetes, light-intensity and moderate-intensity endurance exercises are typically linked to a decrease in glucose levels; however, prolonged exercise increases the risk of hypoglycemia unless there is a decrease in circulating exogenous insulin levels (also known as “insulin on board” (IOB)) and/or carbohydrate intake (34).

### 10.1 Endurance strategy

Large muscle groups move continuously, rhythmically, and repeatedly during endurance exercises, which persist for at least 10 min (e.g., walking, jogging, gardening, and cycling).

*Insulin adjustment:* A specific insulin dose decrease (i.e., bolus and/or basal insulin) is necessary to better manage blood glucose levels in aerobic exercise (e.g., walking, jogging, gardening, and cycling) since it raises the risk of hypoglycemia during the activity. These exercise-specific insulin dose reductions are frequently carried out well in advance of the commencement of the workout to give the body time to lower its insulin levels. Insulin dose reductions (bolus and/or basal insulin) are frequently required during recovery to accommodate the elevated insulin sensitivity that may last for as long as 48 h following the conclusion of exercise (178) (Grade B). Exercise done 2–3 h after receiving a bolus of insulin by CSII or MDI may decrease hypoglycemia if insulin is reduced by 25 to 75%. Regular blood glucose monitoring is necessary while making insulin and carbohydrate changes (2) (Grade C).

Carbohydrate Strategy: In conditions of steady euglycemia, a relatively little amount (10–20 g/h) of carbohydrates may be required during a 30-min bout of moderate-intensity exercise. It is advised to consume more carbohydrates as exercise length and intensity increase, up to 75–90 g/h when working out for more than an hour (34) (Grade C).

Optimization of Performance: The best outcomes are obtained by ingesting 90–144 g/h of CHO in a 2:1 glucose to fructose solution for aerobic workouts that last longer than 2 h. This tactic preserves muscle glycogen while optimizing CHO absorption and oxidation.

### 10.2 High intensity interval training

High-intensity interval training (HIIT) is a popular training technique that minimizes training time commitment while optimizing cardiometabolic adaptations (179). In individuals with type 1 diabetes, this kind of exercise, or training session, is usually linked to reasonable glucose stability; nevertheless, in certain situations (such as fasting or low IOB), it may be linked to either a slight decrease in glucose levels or even an increase in glycemia (180). However, even when glucose levels are trending towards hyperglycemia, this type of exercise might exacerbate misleading symptoms of hypoglycemia in those with T1DM, such as shakiness, sweating, heart palpitations, disorientation, and dizziness (181). Exemplary HIIT activities include cycling, rowing, or running intervals where the effort is close to maximal for brief periods (10 s to 5 min) interspersed with rests or lower intensity effort.

Insulin adjustment: hypoglycemia brought on by exercise can happen during, right after, or even 48 h after exercise, doing so in the evening may result in hypoglycemia while you sleep. Consequently, it is best to avoid working out too late in the evening. Blood glucose levels should be regularly checked before bed, and heavy exertion should be avoided if someone must work out in the evening (182) (Grade C).

CHO Strategy: A single exercise session usually reduces insulin requirements for patients with type 1 diabetes over the course of the following 12 to 24 h. Remarkably, prolonged exercise, such marathon running, might not be linked to improved insulin sensitivity after type 1 diabetes patients’ recuperation and certain types of HIE (intermittent high-intensity exercise) may raise blood glucose levels for a few hours after recovery (183). Reduced insulin and/or higher carbohydrate intake are typically required to avoid hypoglycemia following prolonged (≥30 min), mostly aerobic activity. When circulating insulin levels are low (during fasting or baseline circumstances), about 10–15 g of carbs may help prevent lower blood sugar levels during 30–60 min of mild-to-moderate-intensity aerobic exercise. Similar to the amount of carbohydrates needed to maximize performance in athletes with (184) or without (185) type 1 diabetes, 30–60 g of carbohydrates per hour of exercise may be required for activities carried out with relative hyperinsulinemia (after bolus insulin) (186) (Grade B).

### 10.3 Explosive resistance strategy

“All out” efforts of muscular power and/or speed made for short periods of time (i.e., each effort lasting up to 10–20 s) are what define explosive efforts. In order to produce energy, explosive activities primarily use muscle phosphagen (free adenosine triphosphate and phosphocreatine) and glycolysis. They are also linked to increased sympathoadrenal activation (187), which can lead to an increase in glycemia, especially if this kind of exercise is done alone (188).

Insulin Adjustment: Some patients may have hyperglycemia during vigorous exercise, which may necessitate a correction insulin bolus. This could be due to an immediate stress reaction that reduces insulin sensitivity. The risk of late-onset hypoglycemia may be further increased by this insulin adjustment following exercise. Before they can safely participate in physical activity, diabetic patients undergoing continuous subcutaneous insulin infusion (CSII) or multiple daily injections (MDIs) need to take into account a number of parameters (189). The type of insulin dosage modifications required for exercise (e.g., rapid-acting mealtime insulin vs. long-acting basal insulin) will depend on the insulin management strategy (e.g., MDIs vs. CSII). Understanding the patient’s exercise objectives such as weight loss, fitness, performance and competition, or social interaction is crucial for making decisions.

*Recovery strategy*: Recovery depends on replenishing glycogen stores with quick post-exercise carbohydrate consumption. While some muscle glycogen can be replenished without food consumption, current recommendations suggest consuming 1–1.2 g·kg − 1·h − 1 of carbohydrates within the first 4 h after exercise (77, 78) (Grade C).

## 11 Challenges and future directions

### 11.1 Managing risks of hypoglycemia and hyperglycemia

Hypoglycemia is typically the result of prolonged aerobic exercise that depletes glycogen stores. Reducing insulin levels and increasing carbohydrate consumption before and during extended exercise can help lessen the hypoglycemia effect. Using strength training, quick sprints, or more intense HIIT prior to longer aerobic exercises can also lessen the hypoglycemia response in those with type 1 diabetes (34).

In people with type 1 diabetes, eating more carbs or other macronutrients and adjusting insulin dosage are ways to lower the risk of hypoglycemia during physical activity. The risk of hypoglycemia is decreased by appropriate counselling from a medical professional knowledgeable in insulin dosage schedules and modifications. Furthermore, it has been demonstrated that weight training prior to aerobic exercise lowers hypoglycemia during the latter activity. Lastly, because exercise later in the day may raise the risk of nighttime hypoglycemia, time-of-day modifications are crucial to take into account when administering insulin (190).

Finally, while advising athletes on how to best manage their blood sugar, the doctor needs to be mindful of the potential hyperglycemic response of both high intensity interval training (HIIT) and more strenuous resistance exercise. Individual reactions differ despite these generalizations, and the doctor needs to be ready for hypoglycemia following any activity, particularly when athletes are using insulin (190).

### 11.2 Personalized nutrition approach: emerging technologies and research

Nutrition has a major role in athletic and exercise performance, yet different people react differently to the same foods, nutrients, and supplements. Whether the objective is to maximize physical activity for high performance sports or for health and fitness, this is true for people of all ages, ethnicities, and skill levels. The recent “Nutrition and Athletic Performance” Joint Position Statement by the American College of Sports Medicine, the Academy of Nutrition and Dietetics, and the Dietitians of Canada emphasized the significance of a customized sports nutrition plan, stating that “Nutrition plans need to be personalized to the individual athlete… and take account specificity and uniqueness of responses to various strategies” (77, 78).

Nutrigenomics research is transitioning from fundamental science to practice due to a paradigm change away from the one-size-fits-all group approach and towards individualization. The idea of genotype-based personalized nutrition is not new; there are numerous instances of common (like lactose intolerance) and uncommon (like phenylketonuria) genetic variations that call for certain dietary approaches to be managed. Even while genetic testing is widely utilized in clinical settings, nutrition-focused genetic testing is opening up new avenues for athletes to enhance their health, wellbeing, and athletic performance. In the continuous struggles against harmful supplements (191).

The ability for athletes to use genetic test findings for individualized nutrition in a practical way is the outcome of the scientific information gathered from health and performance studies. Athletes and other active people are increasingly demanding genetic testing for customized nutrition and related performance outcomes, and dietitian-nutritionists, fitness experts, coaches, and other sports medicine practitioners are required to comprehend the available data in this emerging field (192).

### 11.3 Education and support: empowering athletes with diabetes

According to research, professional athletes still maintain inadequate diets and low levels of nutrition awareness despite training in environments that teach them the skills and information necessary to maximize their performance (193). Athletes are vulnerable to contradicting dietary advice from friends, family, teammates, and the internet, much like the general public (194).

Through their sports programs, athletes typically receive structured nutrition education through a variety of modalities, such as digital treatments, interactive activities (such as culinary classes or grocery shop tours), real-time client consultations, and group education sessions (195). The efficiency of various nutrition education approaches in imparting general and sport nutrition knowledge to athletes has not yet been assessed, yet.

In comparison to classroom-based interventions, technology-based (online or mobile) interventions, tailored consultations, shopping trips, and/or cookery classes were under-represented in the assessed intervention studies. It has been demonstrated that supermarket tours and cookery lessons, which can impart useful, skill-based nutrition knowledge, are effective in teaching non-athletic groups how to choose nutritious foods (196). Assessments of these skill-based learning approaches have revealed increased social enjoyment, self-confidence, and acceptability and feasibility (197).

## 12 Conclusion and future recommendations

T1D and T2D athlete’s diets should closely follow the guidelines for treating diabetes, taking into account their energy and nutritional requirements as well as the water loss associated with exercise. The type and severity of diabetes, the regulation of the glucometabolic state, blood sugar levels prior to physical activity, the kind of insulin or other hypoglycemic medications, and their final presumption for participation in sports are also pertinent. In order to engage in physical exercise in a safe and effective manner, it is critical to identify and treat nutritional deficiencies early on by closely monitoring body weight and glycemia. In order to maximize the management of athletes with diabetes for long-term safe and effective dietary therapy, the ongoing support of competent experts is necessary (7).

### 12.1 Future research directions: addressing gaps and challenges

Future researches should focus more on technology driven interventions to enhance and ensure maximum performance of athletes with diabetes.

For example,; the use of continuous glucose monitoring devices. The embedded sensor in continuous glucose monitoring (CGM) devices, which are applied to the arm or belly, measures the amount of glucose in the interstitial fluid either continuously (rt-CGM) or sporadically as needed (198). Additionally, they provide information about the direction, amplitude, length, frequency, and rate of glycemia changes; the data is sent to a recipient, such as a reader device or mobile phone (199). These strategies are useful tools for determining when to start or stop consuming carbohydrates. A standardized glucose target, which is defined as time in range (TIR), time below range, and time over range, is used in the data report to represent overall glycemic management.

One proactive strategy for preventing disordered eating in the active population is to put the targeted nutritional recommendations into practice. It can be beneficial to improve long-term athlete health and performance by recognizing and resolving the particular difficulties that athletes experience, encouraging individualization, and placing an emphasis on a well-rounded and knowledgeable approach to nutrition (86).
